# A beginner's guide to implementation science

**DOI:** 10.1016/j.xjtc.2025.05.005

**Published:** 2025-05-23

**Authors:** Dominique de Waard, Ryan Gainer, Meaghan Sim, Claudia Cote, Philippe Tremblay, Paul Bonnar, Gregory Hirsch

**Affiliations:** a Faculty of Medicine, Dalhousie University, Halifax, Nova Scotia, Canada; b Division of Cardiac Surgery, Halifax Infirmary, Halifax, Nova Scotia, Canada; c IWK Health, Halifax, Nova Scotia, Canada; d Division of Infectious Disease, QEII Health Sciences Centre, Halifax, Nova Scotia, Canada

**Keywords:** implementation science, healthcare, implementation frameworks

**Figure:**
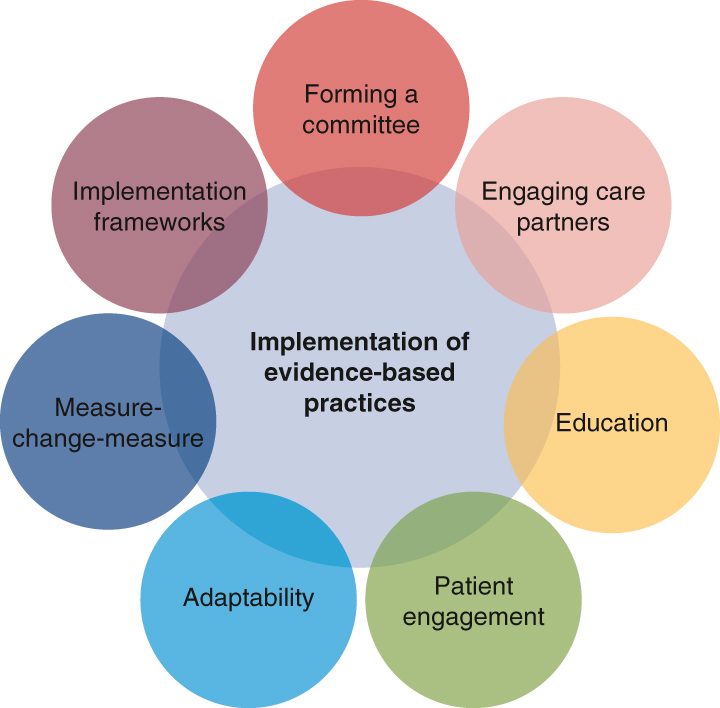
Key factors in successful implementation.

**Central Message**

The field of implementation science is vast and can be difficult to navigate. The majority of clinicians introducing new interventions and/or protocols do not have training in implementation science.

## Approach to Implementation Science

The introduction of new medical interventions takes on average 17 years to integrate into healthcare practice.^1^ Approximately 70% of change efforts do not achieve full implementation.^2^ Implementation science focuses on the adoption, implementation, and sustainability of evidence-based therapies into real-world settings.^3^ Many clinicians lack training in implementation science, which further delays successful adoption of new interventions and protocols. This guide aims to provide a general, quick overview on implementation science and ultimately how to optimize the introduction of new interventions and protocols in a healthcare setting. Additionally, we provide a brief case study outlining our experience with implementation science in cardiac surgery.

### Forming an Implementation Committee

Clear leadership is a well-defined implementation facilitator which is enabled by a dedicated implementation committee.^4^, ^5^, ^6^ An implementation committee is an identified, supportive group that provides space for various implementation partners and/or end users to reflect, learn, and adapt the plan as implementation progresses.^7^ The committee should include individuals from a range of positions who are passionate about the intervention. Involving clinical leads and educators allows for broad reach to many healthcare workers. Often individuals who hold heavy administrative roles are too burdened to focus much time on implementing new interventions. Additionally, engaging individuals who will be responsible for administering or presenting the new intervention is an excellent way to obtain direct, continuous feedback throughout the implementation process.

In our experience, having a dedicated implementation science researcher as a committee member helps lead and sustain the intervention. Funding for such individuals depends on the local organizational structure but may be from a central quality initiative/research program assigned to various projects or more directly by a department/division. An implementation scientist also could be a research assistant or healthcare worker with a focus on implementation and quality improvement. For groups without funding or expertise in implementation, this guide can provide broad steps to implementation science. A dedicated implementation scientist/researcher can guide education sessions, run meetings, and collect quantitative and/or qualitative data. During the implementation period, their role in driving the intervention should decrease over time. If uptake decreases, barriers to independence need to be assessed to understand why independence is lacking.

### Implementation Research Frameworks, Theories, and Models

**Figure 1:**
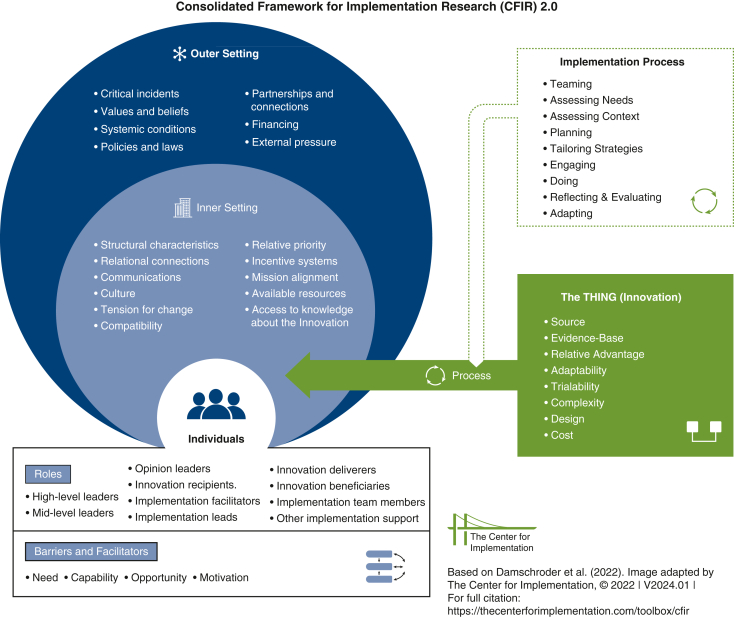
The Consolidated Framework for Implementation Research 2.0 domains and constructs.

Adapted from Damschroderet al.^11^ Image adapted by The Center for Implementation, © 2022. Version: V2024.01. https://thecenterforimplementation.com/toolbox/cfir.

A myriad of resources are available to assist researchers in designing, implementing, and/or evaluating their intervention.^8^, ^9^, ^10^ Many frameworks, theories, and models focus on assessing barriers (ie, factors that obstruct the implementation process) and facilitators (ie, factors that enable the implementation process). When considering which resource to use, the objective of the research and how it would be best guided by a framework should be evaluated.^10^ For example, the Consolidated Framework for Implementation Research (CFIR) is widely used in healthcare settings, and its use is well described in the literature.^11^ The CFIR is comprised of 5 domains: intervention, outer setting, inner setting, individual, and implementation process. Within these domains are a number of constructs, which are well defined by Damschroder and colleagues^11^ and visually adapted by The Center for Implementation (Figure 1). These constructs prompt the researcher to consider relevant barriers and facilitators applicable to their intervention. They can be used at multiple stages including, but not limited to, the study design, implementation process, and surveys or focus group interviews.^11^ Often the implementation or assessment of a new intervention is dependent on the behaviors of those involved. Many frameworks/models are centered around behavior change, including the Theoretical Domains Framework^12^ and the COM-B Model of Behavior and related Behavior Change Wheel.^13^

As mentioned, these are only a few examples of available frameworks. We encourage readers to explore these as well as other frameworks to determine the best fit for their implementation study. For further guidance, a review by Nilsen^8^ provides a broad overview of the numerous approaches in implementation science. Importantly, Nilsen highlights that different frameworks, theories, and models often focus on a particular aspect of implementation and do not provide detailed instructions on how to set up an implementation strategy. In our experience, a framework often provides a helpful guide that we adapt to design, implement, and/or evaluate an intervention. Heiden and colleagues^14^ provide examples of implementation science in surgery and cardiothoracic surgery.

### Engaging Care Partners

A care partner, previously referred to as a stakeholder, is defined as “an individual or a group who is responsible for or affected by health- and healthcare-related decisions that can be informed by research evidence.”^15^ Engaging care partners is an integral part of implementation as it enables collaboration, allows for early adaptation of the implementation strategy, and promotes sustainability.^10^ Examples of care partners include hospital administrators, division/department heads, clinic coordinators, floor managers, educational leads, and patients. After the development of a new standard of care protocol and before its release, it is beneficial to meet with care partners to discuss the protocol as well as any barriers they perceive and to gain their support. Adjustments to the implementation process agreed upon by the implementation committee should be considered. Including such suggested changes empowers care partners to support the change in practice.

### Engaging Patients

Patients and their families are often forgotten during the implementation of a new intervention. Patient engagement in the study design and during implementation can improve uptake, especially if an intervention requires patient and/or family involvement. Providing patients with information and education is a known patient and caregiver preference, and incorporating patient preferences ultimately can lead to increased adherence.^16^^,^^17^ Patient feedback through surveys or focus groups also can be invaluable in improving the understanding of barriers that may be otherwise overlooked by those administering the intervention. Additional engagement strategies, such as patient journey mapping, advisory panels, and co-design workshops, can provide deeper insights into patient experiences, highlight pain points, and identify practical opportunities for tailoring implementation efforts to better meet patient needs.^18^

### Pilot Study

When implementing interventions on a large scale, conducting a pilot study can be beneficial to assess the early success of implementation and identify barriers and facilitators.^19^ A pilot study may include implementing one aspect of the intervention first or the full intervention to a smaller group—for example, initiating an intervention for outpatients only before also approaching inpatients.

### Implementation of the Intervention

Successful uptake of an intervention requires regular communication, adaptability, and frequent education.^4^^,^^14^^,^^17^ It is important to reach all individuals who may be involved in carrying out the intervention so they know what to expect. Providing reasoning/evidence behind the new intervention provides individuals with an understanding of the benefits and increases engagement. Electronic communications through email and educational groups can be sent widely but are insufficient on their own. From our experience, in-person education and educational aids, like posters and pamphlets, yielded higher understanding and uptake.

Education through small and large group presentations is important both at the onset of the intervention and regularly throughout the study period.^17^ In healthcare settings, staffing turnover is a significant barrier to implementation owing to the new staffs’ lack of knowledge of the intervention.^4^^,^^19^ Having educators on the implementation committee can help provide a regular means of educating through updates and general prompts.

In addition to education, automating as much of the process as possible leads to early, successful implementation uptake. With the introduction of electronic medical records (EMR), many orders for interventions or protocols can be integrated into the system. This provides prompting for staff and addresses concerns regarding an individual's lack of knowledge of the intervention.

There are many barriers to implementation in healthcare settings. Common barriers include resistance to change, lack of motivation, poor communication, and cost.^4^, ^5^, ^6^^,^^14^^,^^17^ Resistance to change and lack of motivation may represent major barriers during implementation.^4^, ^5^, ^6^ These barriers can be difficult to navigate, but having care partner support can help enforce change, and using behavior-focused implementation frameworks can encourage motivation. Poor communication, especially about the intervention itself and the implementation strategy, can lead to early discontinuation.^4^^,^^14^ Lack of knowledge and staff turnover are common barriers that can be exacerbated by poor communication.

Finally, cost/resource constraints represent a common barrier in healthcare settings.^4^, ^5^, ^6^^,^^17^ In public systems, the system itself may be resistant to change if there is a large upfront cost associated with the intervention. Presenting clear health and financial benefits when proposing the implementation of an intervention is essential to gain institutional support. Resource-poor settings may need to further adapt their implementation strategy to reduce costs.

### Implementation Setting

An implementation approach including the aforementioned recommendations can be applied to many healthcare settings. Intrinsically, a new intervention is usually easier to implement in a smaller group setting, such as a clinic compared to a hospital.^5^^,^^14^ As mentioned earlier, barriers in implementation science include lack of knowledge and individuals’ motivation to change. In a small group, education on a new intervention is less extensive, and reminders can be easily disseminated. In hospital settings, private institutions may be more motivated to implement a new intervention for financial gain, although some studies have shown no difference in uptake between private and public systems.^17^

Irrespective of the size or type of setting, an organized approach to implementation with a committee and a framework is key to success and sustainability. The use of frameworks in the design process can help the implementation committee identify barriers applicable to their setting early and adapt accordingly.

## Evaluating the Implementation Approach

### Assessing Uptake

To regularly adapt the implementation strategy and to increase uptake of the intervention, a “measure-change-measure” approach is useful. This is similar to the “study” and “act” components of the well-described “plan-do-study-act” method to address issues and assess change.^20^

#### Measure

During the study period, data on successful uptake of the intervention as well as barriers faced leading to lack of uptake should be recorded.

#### Change

Regular assessments of the data throughout the study period allows the implementation team to review areas of weakness and devise strategies to increase uptake. Communicating with individuals involved/affected by barriers is a prudent way to create positive change in the implementation strategy.

#### Measure

After a new approach is implemented, success should continue to be monitored, and the strategy should then be reassessed.

### Focus Group Interviews

Focus group interviews are a prominent feature in implementation science.^21^^,^^22^ They allow individuals involved in the administration/uptake of an intervention a chance to provide feedback on their experience through a semistructured or structured interview process. Focus groups can be convened at different stages of the implementation process to identify barriers and facilitators and/or to evaluate the success of implementation at the end of the study period. Implementation frameworks are useful when creating question guides for focus groups. It is important to consider the real or perceived power imbalances that exist in medicine among healthcare staff.^22^ Focus groups should be designed to optimize engagement from all relevant parties within the context of these power imbalances—for example, separate focus groups with physicians and nurses.

### Surveys

Surveys can be a used in a multitude of ways similar to focus groups. Before introducing an intervention or protocol, a survey can be conducted to identify what other centers/sites do and their implementation strategies. This may help guide the design of a proposal and the implementation process. Surveys also can be used throughout implementation to gather feedback, allowing the committee to address barriers in a timely manner. Finally, surveys can be used to evaluate the success of implementation both qualitatively and quantitatively.

### Defining Success in Implementation Science

Defining success in implementation can be evaluated in various ways, from clinical outcomes and system outcomes to qualitative evaluation. In cardiothoracic surgery, clinical outcomes may include death, major adverse cardiac events, disabling stroke, hospital readmissions, length of stay, reoperation, rate of specific complications (eg, surgical site infection [SSI]), patient reported outcomes, and quality of life scores. There are also a number of frameworks that are used to evaluate implementation, including RE-AIM and PRECEDE-PROCEED.^10^^,^^23^^,^^24^ The concept of implementation outcomes has been popularized by Proctor and colleagues (Table 1).^25^

**Table 1:** Implementation outcomes

Implementation outcome	Definition
Acceptability	The perception that the intervention is considered satisfactory by the implementation care providers
Adoption	The uptake of the intervention
Appropriateness	The intervention's fit, relevance, or compatibility within the setting in which it is being implemented and its ability to address an issue or problem
Cost	The cost associated with implementation of the intervention
Feasibility	The ability of the intervention to be successfully carried out in the respective setting
Fidelity	The extent to which the intervention was delivered as per the original protocol
Penetration	Also referred to as “reach”, the extent to which an intervention was integrated in the intended setting
Sustainability	The maintenance or institutionalization of the intervention over time

Definitions adapted from Proctor et al.^25^

### Long-Term Impact Analysis

Successful uptake of an intervention during a dedicated study period alone does not indicate long term success.^4^ Regular audits of uptake/proper use are vital to ensure longevity. This can be done by ensuring that long-term follow-up is considered in the study design, continuing the measure-change-measure approach outside the study period, assessing the impact on predefined outcomes at different time points, and/or completing a cost analysis. An impact analysis may be approached through quantitative, qualitative, or mixed-methods research.

It is important to have committee members that are involved long-term or continue to introduce new members to ensure ongoing engagement in the intervention, as well as to ensure that long-term impact analyses are done.

## Case Study

At our institution, the cardiac surgery SSI rate was higher than the benchmark rate, and thus we decided to implement *Staphylococcus aureus* screening and decolonization, a strategy well described in the literature to decrease SSIs in cardiac surgery.^26^^,^^27^ Our institution is a tertiary hospital in a publicly funded system without an EMR.

Our implementation committee was motivated to reduce SSI rates and included an implementation scientist, cardiac surgeon, infectious disease physician, health outcomes scientist, and the lead, a cardiac surgery resident with an interest in implementation science. We used CFIR to help guide our implementation process. By reviewing the CFIR domains and constructs, we were able to anticipate early barriers—including lack of individual knowledge, supply availability, timing delays associated with screening time—and adjusted our implementation strategy accordingly. CFIR was also used to develop questions for care partner meetings and focus groups.

We approached care partners to propose the intervention, outline the implementation strategy, and understand/adjust for any perceived barriers. Care partners included the division heads of cardiology and cardiac surgery, nursing managers of cardiology and cardiac surgery floors, and the microbiology lab director.

Before the rollout of *S. aureus* screening and decolonization, educational nursing leads were approached for information dissemination and to organize education sessions. An informative email was sent to the divisions of cardiac surgery and cardiology before and at the start of implementation. Educational resources were developed, and regular educational sessions were held with staff during the 1-year study period, as staffing turnover was highlighted as a recurrent barrier.

Throughout the study period, quantitative and qualitative data highlighted barriers such as staffing turnover, lack of knowledge, and timing constraints. We addressed these barriers by creating preprinted orders, continuing education sessions, involving educational leads in the implementation process, and having the microbiology lab call with positive screening results.

Over the 1-year study period, we saw an increase in uptake of the intervention. Screening implementation was very successful, while decolonization uptake was slower because of barriers. Long-term quality assessments showed ongoing increased uptake of decolonization over time. Along with the introduction of other SSI reduction strategies, we saw a decline in SSIs over time.

## Conclusions

Implementation science provides a structured approach to assessing the uptake of evidence-based therapies in real-world settings. Implementation science frameworks, models, and theories serve as a guide to assess barriers and facilitators of an implementation strategy. They can assist researchers, clinicians, and decision makers during the study design, implementation, and evaluation stages. Other basic principles of implementation science include developing a research protocol, forming a committee, engaging in regular education, seeking regular feedback, and adapting the implementation strategy to increase uptake. Although these principles seem basic, an organized approach to the implementation of evidence-based treatments/protocols can help ensure adoption and sustainability.

## Conflict of Interest Statement

Dr de Waard serves as a paid consultant for Johnson & Johnson MedTech. Dr Tremblay has received consulting/speaker fees from Lung Bioengineering, Atricure, and Abiomed. Dr Bonnar is actively doing research with AXDEV Group and receives Canadian Institute of Health Research grants. All other authors reported no conflicts of interest.

The *Journal* policy requires editors and reviewers to disclose conflicts of interest and to decline handling or reviewing manuscripts for which they may have a conflict of interest. The editors and reviewers of this article have no conflicts of interest.
